# The Impact of Cardiovascular Risk Factors on the Incidence, Severity, and Prognosis of Sudden Sensorineural Hearing Loss (SSHL): A Systematic Review

**DOI:** 10.7759/cureus.58377

**Published:** 2024-04-16

**Authors:** Anna-Maria Papadopoulou, Sotirios Papouliakos, Petros Karkos, Konstantinos Chaidas

**Affiliations:** 1 Department of Anatomy, School of Medicine, National and Kapodistrian University of Athens, Athens, GRC; 2 Department of Otolaryngology, General Hospital of Athens "G. Gennimatas", Athens, GRC; 3 Department of Otolaryngology - Head and Neck Surgery, University General Hospital of Thessaloniki (AHEPA), Thessaloniki, GRC; 4 Department of Ear, Nose, and Throat, School of Medicine, Democritus University of Thrace, University Hospital of Alexandroupolis, Alexandroupolis, GRC

**Keywords:** risk factors, vascular etiology, metabolic etiology, atherosclerosis, sudden sensorineural hearing loss

## Abstract

Sudden sensorineural hearing loss (SSHL) is believed to be mainly idiopathic since the cause is not usually identified. Several recent studies have examined the role of cardiovascular risk factors in this disease. The aim of this systematic literature review is to investigate the possible association between acquired and inherited cardiovascular risk factors and the incidence, severity, and prognosis of SSHL. A systematic review was conducted according to the Preferred Reporting Items for Systematic Reviews and Meta-Analyses (PRISMA) guidelines. A search of the PubMed database for the period between February 2010 and January 2023 was performed in order to retrieve eligible articles. The analytic cohort included 24 studies. Overall, this systematic review includes a total of 61,060 patients that were encompassed in these studies. According to most studies, the prevalence of dyslipidaemia, diabetes, and ultrasound indices of atherosclerosis was significantly higher in SSHL patients compared to controls. On the other hand, obesity, hypertension, and smoking did not seem to influence the risk of SSHL. Most studies suggest the presence of a correlation between a high cardiovascular risk profile and the risk of developing SSHL. The theory of microvascular impairment in the development of SSHL is indirectly supported by the findings of this review.

## Introduction and background

Sudden sensorineural hearing loss (SSHL) is usually described as a hearing loss of at least 30 dB, over three sequential pure tone frequencies, developing within a three-day period [1]. The estimated annual incidence is approximately 5-30 per 100,000 people, with equal gender and affected side distribution [2,3]. SSHL is an otologic emergency and requires immediate treatment [4]. Although approximately 32%-45% of the patients recover fully or partially, a significant percentage suffers from permanent hearing loss [5,6]. Notably, 13% of patients who recover spontaneously will predictably experience a recurrent episode of SSHL [7]. Other SSNHL-associated symptoms include tinnitus, aural fullness, sound distortion, and vertigo [1]. SSHL is thought to be mostly idiopathic since the cause is rarely identified, which influences the planning of preventive and therapeutic strategies [8,9]. Several factors affecting the function of the cochlea, the cochlear nerve, the system that transmits sound from the cochlea to the brain, or other portions of the central auditory nervous system are considered to induce SSHL [1]. Microcirculation disturbance, intracochlear membrane rupture, viral infections, trauma, neoplasms, and autoimmune and genetic factors have been proposed as possible theories explaining the pathogenesis and onset of SSHL [10,11]. The vascular theory proposes that SSHL is a result of acute vascular haemorrhage, occlusion by emboli, vascular disease, vasospasm, or changes in blood viscosity with subsequent hypoxia and ischaemia of inner ear structures [1,12,13]. Cochlear hair cells require high energy for their metabolic activity, and constant blood flow is vital for hypoxic or ischaemic damage prevention [12,14]. The labyrinthine artery, a terminal branch of the anterior inferior cerebellar artery, provides blood supply to the inner ear, an “end organ”, with a deficiency of collateral circulation [10]. This is the most popular theory, although the occlusion of such a microcirculation within the temporal bone cannot be detected by any objective test [15]. Consequently, several recent studies have focused on the role of atherosclerosis and its associated risk factors, such as diabetes, hypertension, obesity, hypercholesterolaemia, cerebrovascular disease, and smoking in SSHL [1,14]. Moreover, the association between cardiovascular risk factors and SSHL treatment outcomes remains uncertain.

The aim of this systematic review is to investigate the potential association between acquired and inherited cardiovascular risk factors and the incidence, severity, and prognosis of SSHL.

## Review

Methods

The Pubmed database was searched for relevant articles for the period between February 2010 and January 2023. Only articles published since 2010 were selected since we intended to focus on the latest research and not on outdated and historical knowledge. The search strategy included the terms “cardiovascular risk factors” or “atherosclerosis” or “thromboembolic risk factors” and “sudden sensorineural hearing loss” or “sudden deafness”. Titles of interest were reviewed by abstract, and if considered relevant, they were included for full-text review. Review articles, as well as case reports, were excluded, whereas all prospective and retrospective studies were suitable for this systematic review. Articles with full text available in English, French, and German were considered eligible. Finally, reference lists of all selected articles were manually assessed so that all potential additional relevant articles were detected (“snow-ball” procedure). The systematic review was performed according to the Preferred Reporting Items for Systematic Reviews and Meta-Analyses (PRISMA) guidelines.

Results

Article Selection and Study Demographics

**Figure 1:**
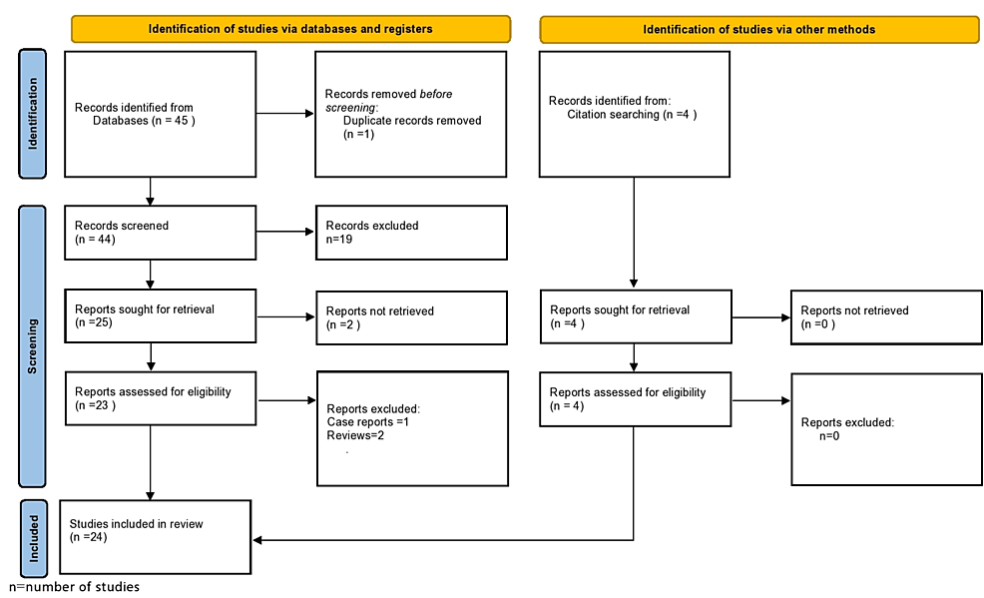
Flowchart of the search strategy

Following the screening of titles and abstracts, 42 articles were retrieved by the search strategy and were selected for full-text evaluation. The analytic cohort included 24 studies that were considered eligible. Overall, this systematic review includes a total of 61,060 patients that were encompassed in these studies. The search strategy is presented in Figure 1.

Main Findings

Correlation between cardiovascular risk factors and SSHL: The majority of collected studies were case-control studies comparing the existence of risk factors for cardiovascular disease between patients with SSHL and normal controls.

According to most studies, which also included the largest number of participants, total cholesterol values were significantly higher in SSHL patients in comparison with controls (Table 1). More specifically, the SSHL group was characterized by significantly higher rate of low levels of high-density lipoprotein-cholesterol (HDL-C). On the other hand, most authors came to an agreement that low-density lipoprotein-cholesterol (LDL-C) levels were similar in patients and controls. Moreover, the results of studies containing the larger group of patients showed that elevated triglyceride (TG) levels were significantly correlated with the prevalence of SSHL. Plasma levels of apolipoprotein B were measured in two studies, and in both of them, statistical significance was reached when the two groups were compared [13,16]. Kaneva et al. also proved that higher measurments of the atherogenic index were significantly related to an increased risk of idiopathic SSHL [13]. On the contrary, a recent retrospective study did not find a significant correlation between dyslipidaemia and SSHL [17].

**Table 1:** Comparison of lipidaemic profiles in SSHL patients vs controls (p-value) * p<0.05; ** p<0.01; *** p<0.001 SSHL: sudden sensorineural hearing loss, HDL: high-density lipoprotein, LDL: low-density lipoprotein TGL: triglycerides apoB: apolipoprotein B, CCS: case-control study, RCCS: retrospective case-control study, PCCS: prospective case-control study

Study, year	Study design	Number of participants	Total cholesterol (SSHL vs controls)	HDL (SSHL vs controls)	LDL (SSHL vs controls)	TGL (SSHL vs controls)	apoB (SSHL vs controls)
Aimoni et al., 2010 [18]	CCS	412	0.006	-	-	0.25	-
Cadoni et al., 2010 [3]	CCS	86	<0.001***	-	-	-	-
Ciccone et al., 2012 [1]	CCS	58	0.04*	0.74	0.02*	0.77	-
Yasan et al., 2012 [19]	CCS	250	-	0.8	0.4	0.3	-
Weng et al., 2013 [16]	RCCS	500	<0.01**	0.44	<0.01**	0.574	0.03*
Chien et al., 2015 [20]	RCCS	362	-	<0.01*		<0.01*	-
Lee et al., 2015 [21]	CCS	1296	<0.001***	<0.001***	0.12	0.003**	-
Passamonti et al., 2015 [22]	CCS	532	<0.05	-	-	-	-
Chung et al., 2016 [4]	PCCS	108	0.49	0.48	0.85	0.055	-
Rajati et al., 2016 [23]	CCS	60	0.38	0.16	0.28	0.69	-
Fasano et al., 2017 [6]	PCCS	208	0.22	0.25	0.04*	-	-
Kaneva et al., 2019 [13]	CCS	51	0.14	0.08	0.27	0.2	0.02*
Ho et al., 2020 [8]	CCS	80	0.688	0.994	0.117	-	-
Jalali et al., 2020 [14]	CCS	324	0.5	<0.001***	0.126	0.007	-
Wang et al., 2020 [2]	CCS	1296	<0.001***	0.0002***	0.09	0.003**	-

Additionally, according to most and largest studies, statistically significantly higher levels of fasting blood glucose were encountered in SSHL patients in comparison with controls (Table 2). Similarly, patients with SSHL had higher levels of glycated haemoglobin (HbA1C) in one study [6]. Interestingly, almost all authors who compared the history of diabetes mellitus between patients and controls did not find statistically significant difference (Table 2). Nevertheless, a large retrospective study, including 53,112 patients, proved that there was a 1.54-fold higher incidence of SSHL in the diabetic group compared to the non-diabetic group [18]. The severity of diabetes, as well as coronary heart disease or retinopathy comorbidities in diabetic patients, also increased the risk of SSHL development [24]. In addition, Tsuzuki et al. proved that diabetes was related to higher thresholds in SSHL [25].

**Table 2:** Comparison of diabetic profiles in SSHL patients vs controls (p-value) * p<0.05; ** p<0.01; *** p<0.001 SSHL: sudden sensorineural hearing loss, CCS: case-control study, RCCS: retrospective case-control study, PCCS: prospective case-control study RCA: retrospective chart analysis

Study, year	Study design	Number of participants	Fasting blood glucose (SSHL vs controls)	Diabetes (SSHL vs controls)
Aimoni et al., 2010 [18]	CCS	412	-	0.03*
Monsier et al., 2011 [11]	CCS	275	-	>0.05
Ciccone et al., 2012 [1]	CCS	58	0.46	0.7
Yasan et al., 2012 [19]	CCS	250	0.03*	-
Chien et al., 2015 [20]	RCCS	362	<0.01**	0.2
Chung et al., 2016 [4]	PCCS	108	-	0.09
Rajati et al., 2016 [23]	CCS	60	0.63	-
Fasano et al., 2017 [6]	PCCS	208	<0.01**	-
Ho et al., 2020 [8]	CCS	80	-	0.402
Jalali et al., 2020 [14]	CCS	324	0.9	0.07
Tsuzuki et al., 2022 [25]	RCA	762	-	<0.05*
Lenkeit et al., 2023 [17]	CCS	223	-	>0.05

As far as obesity is concerned, different studies yield contradictory results. Most of them found no difference in the body-mass index (BMI) between patients and controls (Table 3). Only one study demonstrated a significant correlation between increased BMI and the prevalence of SSHL and its prognosis [24]. Furthermore, Chien et al. proved that waist circumference differed significantly between the two groups (Table 3) [20]. They also showed that there was a 3.54-fold increased risk of sudden sensorineural hearing loss in patients with metabolic syndrome compared with those without (Table 3) [20].

**Table 3:** Comparison of BMI, waist circumference, and metabolic syndrome in SSHL patients vs controls (p-value) * p<0.05; ** p<0.01; *** p<0.001 SSHL: sudden sensorineural hearing loss, CCS: case-control study, RCCS: retrospective case-control study, PCCS: prospective case-control study, BMI: body mass index

Study, year	Study design	Number of participants	BMI (SSHL vs controls)	Waist circumference (SSHL vs controls)	Metabolic syndrome (SSHL vs controls)
Monsier et al., 2011 [11]	CCS	275	0.4	-	-
Ciccone et al., 2012 [1]	CCS	58	>0.05	-	-
Chien et al., 2015 [20]	RCCS	362		<0.01**	<0.05*
Lee et al., 2015 [21]	CCS	1296	>0.05	-	-
Chung et al., 2016 [4]	PCCS	108	-	0.52	-
Rajati et al., 2016 [23]	CCS	60	0.07	-	-
Jalali et al., 2020 [14]	CCS	324	0.052	-	>0.05
Wang et al., 2020 [2]	CCS	1296	0.004**	-	-
Lenkeit et al., 2023 [17]	CCS	223	>0.05	-	-

According to most authors, a history of hypertension did not influence the risk of SSHL (Table 4). Nonetheless, Monsier et al. [11] pointed out that systolic blood pressure was significantly higher in patients (p=0.003), and Chien et al. [20] confirmed this finding, demonstrating higher measurements of both systolic and diastolic blood pressure. Interestingly, Chung et al. measured brachial-ankle pulse wave velocity (BaPWV) and found that it was higher in the SSHL group compared to the control group (p=0.036) [4].

**Table 4:** Comparison of hypertension, smoking, and CIMT in SSHL patients vs controls (p-value) * p<0.05; ** p<0.01; *** p<0.001 SSHL: sudden sensorineural hearing loss, CCS: case-control study, RCCS: retrospective case-control study, PCCS: prospective case-control study, CIMT: carotid intima-media thickness

Study, year	Study design	Number of participants	Hypertension	Smoking	CIMT
Aimoni et al., 2010 [18]	CCS	412	0.9	0.7	
Monsier et al., 2011 [11]	CCS	275	0.003**	>0.05	
Ciccone et al., 2012 [1]	CCS	58	>0.05	0.5	0.61
Chien et al., 2015 [20]	RCCS	362	<0.05*	0.77	
Chung et al., 2016 [4]	PCCS	108	0.036 *		
Rajati et al., 2016 [23]	CCS	60	0.045*	0.93	0.002**
Mutlu et al., 2017 [12]	RCCS	90			<0.001***
Ho et al., 2020 [8]	CCS	80	0.151	0.512	0.022*
Jalali et al., 2020 [14]	CCS	324	0.033*	0.93	
Wang et al., 2020 [2]	CCS	1296	0.12		
Lenkeit et al., 2023 [17]	CCS	223	>0.05		

All studies came to an agreement that patients and controls did not differ significantly concerning the smoking prevalence (Table 4). On the contrary, Monsier et al. highlighted that patients had a higher incidence of personal and family history of cardiovascular disease than controls [11]. Other studies proved that hyperhomocysteinemia, high factor VIII, and deficiencies of antithrombin, protein C, or S increase the risk of SSHL [6,22,26]. Ballesteros et al. also described a higher prevalence of the 807T thrombophilic polymorphism of platelet glycoprotein Ia/IIa in patients with SSHL [27].

Furthermore, many authors measured ultrasound indices of subclinical atherosclerosis. The majority of studies showed that SSHL patients exhibited a significantly higher average carotid intima-media thickness (CIMT) than that in the control group (Table 4). In fact, Mutlu et al. found that parameters such as resistive index, peak systolic velocity, and blood flow of both the carotid and vertebral system differed significantly between the two groups [12]. Finally, significantly lower flow-mediated dilation (FMD) of the brachial artery values were found in SSHL patients compared with controls, according to Ciccone et al., which was highlighted by the multiple logistic regression. They concluded that the reduction in hearing in SSHL patients could be independently expected by a lower FMD value [1].

Prognostic factors of recovery: The initial hearing threshold has been proven to be the main prognostic factor of recovery, according to most studies [1,4,11,19]. One study also found that the hearing outcome may be influenced by the initial hearing loss pattern [20]. As far as cardiovascular risk factors are concerned, Passamonti et al. associated arterial hypertension, hyperlipidaemia, smoking, and diabetes with a poor SSHL clinical outcome [22]. Interestingly, patients with a higher Framingham risk score had poorer hearing recovery, which could make this score a useful predictive tool concerning outcomes of idiopathic sudden sensorineural hearing loss [28]. Moreover, Jung et al. found that patients with metabolic syndrome had a lower rate of recovery compared to those without and that patients with more than four components of metabolic syndrome had poorer prognoses [29]. Additionally, patients with poor hearing prognosis had significantly higher internal and common carotid pulsatility and resistive indexes compared to those with good prognosis, while the variables of the vertebral artery were not associated with the prognosis of SSHL [20]. Conversely, another study pointed out that groups with low and high IMT had similar severity of hearing loss and treatment outcomes [8]. Finally, baPWV and a history of hypertension and diabetes initially seemed to be related to hearing recovery, according to Chung et al., but this was not established by multivariate analysis [4].

Discussion

SSHL is a well-known, but still not well-defined, clinical entity. The need for prevention and early detection has encouraged investigation of its pathogenesis, as well as its related diseases, according to the current otologic literature [29].

The inner ear is regarded as an organ with a high metabolism. Cochlear outer hair cells resemble cardiac myocytes concerning their motility; thus, a great amount of energy is required for their contraction [30]. As a result, impaired blood perfusion and microvascular damage of the cochlea are possibly important causes of SSHL [30]. Notably, the cochlea has very limited tolerance to ischaemia; according to an experimental study, 60 seconds of anoxia impairs the action potential, and one hour of vessel obstruction causes permanent damage to cochlear function [31]. Several other possible theories have been proposed to explicate the vascular theory in SSHL pathogenesis. Firstly, the abrupt onset of the disease resembles ischaemic vascular diseases such as myocardial infarction, transient ischaemic attacks, or amaurosis fugax [27]. Moreover, histopathological damage has been found to the spiral ganglion cells and the organ of Corti due to vascular obstruction [32]. In addition, spinal cord manipulation has been suggested to induce SSHL, possibly through injury of vertebrobasilar vessels and interestingly, sympathetic ganglion block in the neck appears to be beneficial by improving inner ear circulation disturbances [23,33]. Finally, the blood supply of the cochlea depends on a single posterior terminal branch [34]. Genetically established susceptibility for thrombosis or risk factors for cardiovascular disease can cause microcirculation alteration in the inner ear [35]. Unfortunately, auditory pathway damage cannot be easily detected [8]. Only experimental studies and indirect clinical arguments could endorse these theories since the inner ear is located in the petrous bone [11]. Many studies have assessed the potential role of various cardiovascular and thrombophilic factors in the development of SSHL, in an attempt to distinguish possible risk factors that are connected to impaired vascular perfusion [18]. Most of them indicate that the risk factors of SSHL coincide with those of cardiovascular disease [16,36].

Additionally, an increased rate of cardiovascular disease has been demonstrated among patients with SSHL [24]. A population-based cohort study postulated that myocardial infarction might be independently associated with SSHL [37]. Other studies confirm that SSHL patients are at higher cardiovascular risk, while others suggest that atherosclerosis of the vessels in the inner ear precedes atherosclerosis in other larger vessels [1,38].

We conclude that dyslipidaemia, characterized by hypercholesterolaemia and/or triglyceridaemia, is a risk factor for SSHL. Dyslipidemia can induce cochlear ischaemia through increased blood viscosity, cochlear vessel atherosclerosis, and impairment of erythrocyte oxygen-carrying capacity [36]. According to an experimental study, cholesterol also affects the structure and function of outer hair cells at a molecular level [39]. Furthermore, clinical and experimental studies have suggested that correction of hyperlidaemia tends to improve hearing in SSHL patients and that inner ear damage as well as hearing loss might be prevented by statins [40,41]. In fact, Wang et al. proposed that high-risk individuals with SSHL could be initially screened for serum non-HDL-C [2].

Diabetes may also result in SSHL through cochlear microangiopathy and neuropathy of the spiral ganglion and the eighth cranial nerve [37]. Studies in human temporal bones have repeatedly demonstrated that diabetes is associated with cochlear microangiopathy, whereas electrophysiological studies further revealed retrocochlear pathologies [42].

Hyperhomocysteinemia, high levels of factor VIII, and other acquired and inherited risk factors for thrombophilia have also been encountered in SSHL patients [6,22]. It has been shown that homocysteine harms endothelial cells, reduces vessel flexibility, and negatively affects hemostasis [43]. The formation of large thrombi on damaged endothelium can be stimulated by high factor VIII levels [43].

Obesity is thought to be an independent risk factor for age-related hearing loss, and higher BMI has been correlated with more severe sensorineural hearing loss [29]. However, it is reported by most studies that obesity is not a risk factor for SSHL.

Similarly, smoking and hypertension are well-known risk factors for cardiovascular disease [11]. However, their prevalence did not differ significantly between SSHL patients and controls, according to most of the collected studies.

There is still controversy concerning the prognosis of SSHL according to the literature. Treatment outcomes can be affected by audiological factors, such as the severity of hearing loss initially, the interval between the onset of hearing loss and the beginning of treatment, or coexisting vertigo [8]. Some authors suggest that the existence of cardiovascular risk factors may have an effect on the hearing outcome in SSHL, while others do not [11,44].

Many studies yield contradictory results regarding the association between cardiovascular risk factors and SSHL. The discrepancies may originate from flawed control groups and population-based cohort studies would elucidate these disagreements. Moreover, the use of medication, such as statins or antidiabetic drugs, could partly explain the different findings. Finally, Kaneva et al. emphasized that studies not showing a correlation between blood lipid levels and SSHL did not evaluate atherogenicity in detail and the lipid profile was assessed by conventional lipid indices [13].

## Conclusions

Future large-scale, prospective, and population-based cohort studies on SSHL incidence in patients with cardiovascular risk factors could confirm whether atherosclerosis increases the risk of SSNHL development and clarify the role of microvascular impairment and cardiovascular risk factors. In the meantime, we suggest that screening for traditional cardiovascular risk factors could be performed in SSHL patients so that appropriate preventive and therapeutic strategies can be implemented. Conversely, early markers of endothelial dysfunction, such as CIMT and flow-mediated dilatation (FMD), could help in identifying patients at a high risk for SSHL.

Most studies suggest that a patient profile with a high cardiovascular risk is independently and significantly connected with the risk of SSHL. It can be further proposed that metabolic and vascular impairment has a key role in the pathogenesis of SSHL. As a result, the theory of microvascular impairment in the development of SSHL is indirectly supported by the findings of this review.
